# Spigot in pneumothorax cavity: A rare complication of endobronchial Watanabe spigot

**DOI:** 10.1002/rcr2.823

**Published:** 2021-08-05

**Authors:** Shion Miyoshi, Kyohei Kaburaki, Hajime Otsuka, Kazuma Kishi

**Affiliations:** ^1^ Department of Respiratory Medicine Toho University Omori Medical Center Ota‐Ku Japan; ^2^ Division of Chest Surgery, Department of Surgery Toho University School of Medicine Ota‐Ku Japan

**Keywords:** bronchial occlusion, bronchoscopy, complication, pneumothorax, spigot

## Abstract

Spigot dropping is a rare but an intractable complication, because it is difficult to salvage a dropped spigot from the thoracic cavity. If the pneumothorax with a large fistula is recurrent, replacement with a larger size spigot may be recommended.

## Graphical abstract

Spigot dropping is a rare but an intractable complication of bronchial occlusion because it is difficult to salvage a dropped spigot from the thoracic cavity. If the pneumothorax with a large fistula is recurrent, replacement with a larger size spigot may be recommended.

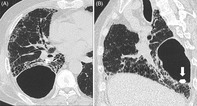

## CLINICAL IMAGE

**FIGURE 1:**
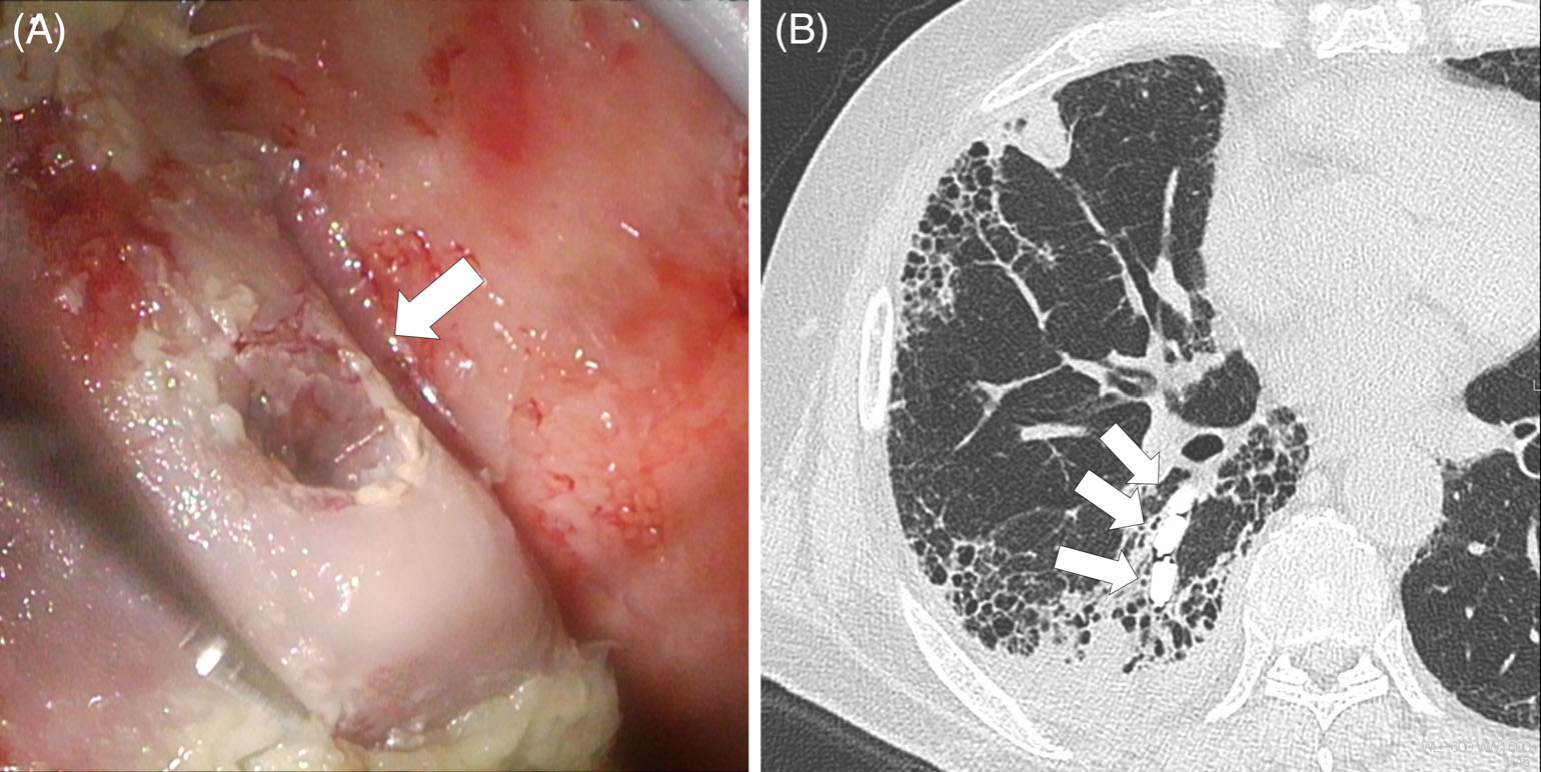
Pyopneumothorax treated with thoracoscopic surgery and bronchial occlusion using the endobronchial Watanabe spigot (EWS) technique. The intraoperative findings showed a large amount of white coat and a fistula of 1 cm in diameter at S6 (A, arrow). Finally, three EWSs remained in his bronchus and his condition improved (B, arrows)

**FIGURE 2:**
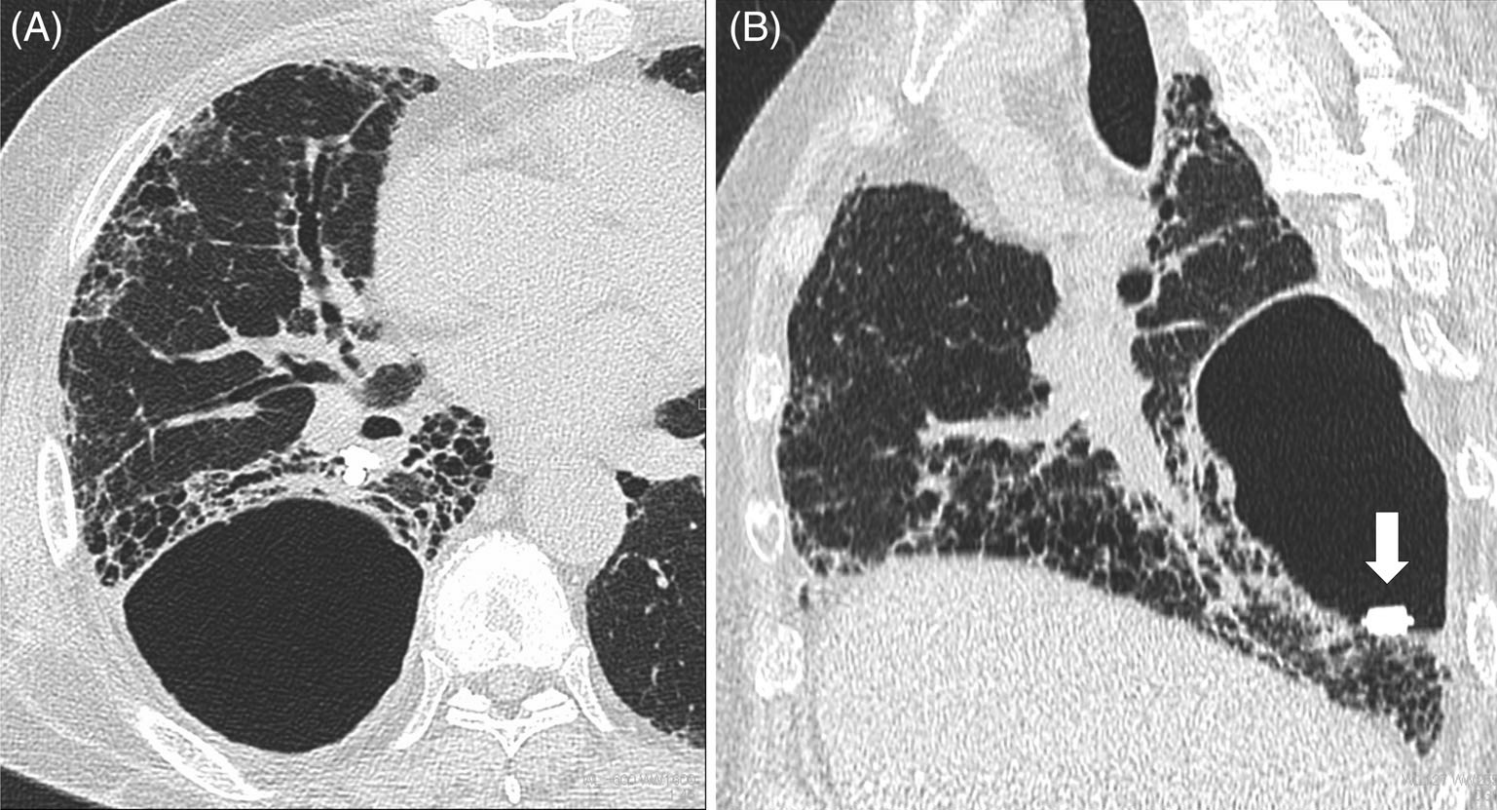
Recurrent pneumothorax and the endobronchial Watanabe spigot (EWS) dropout through the pulmonary fistula into the thoracic cavity. After 6 months of treatment, his pneumothorax was recurrent, but no respiratory failure developed because the lung partially adhered to the chest wall (A). The enlargement of the pneumothorax cavity caused an EWS to drop into the thoracic cavity (B, arrow)

A 57‐year‐old man suffering from rheumatoid vasculitis and interstitial pneumonia who received immunosuppressants underwent flexible bronchoscopy as part of an evaluation of a cavitary lesion in his right lung. After flexible bronchoscopy, he developed pyopneumothorax. Despite chest tube drainage, he suffered prolonged air leaks. He was treated with thoracoscopic surgery (Figure 1A), pleurodesis and bronchial occlusion using the endobronchial Watanabe spigot (EWS) technique for prolonged pneumothorax (Figure 1B),^1^ and his condition improved. Three EWSs remained in his bronchus. *Aspergillus* species were detected by pleural empyema culture, so an antifungal drug was administered. After 6 months, his pneumothorax was recurrent, but no respiratory failure developed because the lung partially adhered to the chest wall (Figure 2A). The enlargement of the pneumothorax cavity caused an EWS to drop into the thoracic cavity (Figure 2B). Depending on the clinical course, surgical removal of the dropped EWS is being considered. We presented the first case of EWS dropout through the pulmonary fistula. Several cases of migration of EWS have been reported but the dropout through the fistula is a rare complication.^2^ If the pneumothorax with a large fistula is recurrent, replacement with a larger size EWS may be recommended.

## CONFLICT OF INTEREST

None declared.

## ETHICS STATEMENT

Appropriate written informed consent was obtained for publication of this case report and accompanying images.

## AUTHOR CONTRIBUTIONS

Shion Miyoshi contributed substantially to the writing of the manuscript and medical examination. Kyohei Kaburaki and Kazuma Kishi contributed substantially to critical review and medical examination, and Hajime Otsuka contributed to the surgical treatment. All authors reviewed and approved the final version of the manuscript.

## References

[1] Watanabe Y , Matsuo K , Tamaoki A , Komoto R , Hiraki S . Bronchial occlusion with endobronchial Watanabe spigot. J Bronchology Interv Pulmonol. 2003;10:264–7.

[2] Kaneda H , Minami K , Nakano T , Taniguchi Y , Saito T , Konobu T , et al. Efficacy and long‐term clinical outcome of bronchial occlusion with endobronchial Watanabe spigots for persistent air leaks. Respir Investig. 2015;53:30–6. 10.1016/j.resinv.2014.09.002 25542601

